# Structure–function relationships across scales: implications for atlases

**DOI:** 10.3389/fnins.2025.1750272

**Published:** 2026-01-14

**Authors:** R. Todd Constable

**Affiliations:** Department of Radiology and Bioimaging Science, Yale University School of Medicine, New Haven, CT, United States

**Keywords:** atlas, atlases, brain, cells, functional imaging, organization, structure

## Abstract

Neuroscience aims to understand how the structural and functional organization of the brain relates to behavior. Structural studies at the cellular level establish the neurobiological framework for understanding function. Structure is only part of the story however, with molecular and functional information needed for a comprehensive view. At the mesoscopic level, functional regions are defined by ensembles of neurons that work together. This perspective reviews the relationship between structural and functional organization across scales and aims to provide a common language for neuroscientists working at any level. While it is unequivocal that structure constrains function at the cellular level, understanding function at the meso- and macro-scopic scales is much more complicated involving ensembles of neurons and their dynamic interactions. Recognition of these scale differences is essential for advancing representational models in the field.

## Highlights

- Origin and basis of brain atlases are discussed.
- The underlying assumptions of atlases, and their use, are reviewed.
- At the microscopic scale, structure defines function, but functional integration tends to be rich – neurons are densely connected.
- At the mesoscopic scale structural organization is fixed and sparse while functional organization is complex, rich, and flexible.
- Atlas boundaries correspond poorly to functional boundaries when function is defined as ensembles of neurons working together.
- Atlas nodes tend to be thought of as cohesive units whereas there is significant functional specialization within and across regions—the tens of millions of neurons in a node do not all do the same thing.
- There is a flexible functional organization sitting on top of a fixed anatomic infrastructure.

## Outline

1. Introduction
2. Early measures linking gross anatomy to function
3. Microscopic techniques and the complexity of a single cell
4. The emergence of atlases: Grouping cells into regions
5. Lesion studies for functional localization
6. Mesoscopic structural imaging – Anatomic features and DTI
7. Mesoscopic functional Imaging - Task based localization
8. Mesoscopic functional connectivity – The connectome(s) and parcellation(s)
9. Small world properties and flexible functional considerations
10. Dynamics and functional flexibility at the cellular level
11. Dynamics and functional flexibility at the macroscopic level
12. Reconciling dynamics in the presence of fixed anatomy
13. Linking functional organization to behavior yields extensive whole-brain networks
14. Linking structure and functional at the mesoscopic level
15. Caution in how brain atlases are applied and other considerations
16. Moving forward/how to accommodate flexible functional organization
17. Summary

## Introduction

A primary goal of neuroscience is to understand how the brain works in the context of its structural and functional organization. This perspective focuses on the relationship between structure and function across scales and their link to brain atlases. Advances in *in vivo* cellular mapping and the rapid growth in connectivity studies has led to the creation of many atlases reflecting a wide range of properties.

In the following sections, we explore structure–function relationships from the cellular-level through meso- to macro-scopic neuroimaging scales. Recent work in cellular-neuroanatomy, highlights the brain’s complexity at both the structural and functional levels. The quest for brain atlases is discussed, in terms of the relationship to cellular-level anatomy, and the grouping of cells into regions, and the assumptions atlases make with respect to function. At the mesoscopic scale, structural and functional imaging techniques are discussed, in terms of their roles in creating atlases. Small world properties of the brain and the presence of functional dynamics emerging from a fixed anatomic infrastructure are covered. The assumptions made with atlases are reviewed and as is how these assumptions impact usage. We conclude with recommendations for advancing models linking structure and function across scales.

This is an exciting time in neuroscience research, with increasingly sophisticated efforts to link measures across different scales. Each day brings new advancements in understanding the relationship between structure and function, providing mechanistic insights for understanding function. It is critical for neuroscientists working at various scales to communicate effectively and understand the relationships between measures at all scales. This review is intended to facilitate meaningful conversations across scales and measures.

### Early measures linking gross anatomy to function

Early methods for localizing function came from brain injury studies of patients such as Phineas Gage (Hayward, 2002) and HM (Squire, 2009). Broca’s area (Berker et al., 1986) was identified through lesion studies, and Wernicke’s area was defined in a similar fashion (Binder, 2015). The disruption of function due to a local lesion typically infers some critical functional role to that brain region. Observations of such deficits continues today with lesions caused by tumors, strokes, and other mechanisms (Adolphs, 2016). Electrophysiological instruments have led to *in vivo* means of mapping function locally. The use of electrodes during surgical intervention in the awake patient allows temporary lesions to be induced and the impact on patient behavior observed (Awad et al., 1991). Such studies are necessarily limited in scope but provide a means to directly link brain to behavior. These studies are not sufficient to construct brain atlases however because of the poor definition of the lesion volume, or because the lesion volume may span multiple functional regions through a mass effect or infiltration. In addition, factors such as age at onset and growth rate can affect local and distant functional through loss of function and/or compensatory reorganization. Finally in many such patients it can be difficult to assess behavioral deficits post-lesion, and pre-lesion data is often not available.

### Microscopic techniques and the complexity of a single cell

In the earliest days of neuroscience all that could be measured directly was structure, either through direct dissection at the macroscopic level or through the study of individual cells at the microscopic level. The initial division of the cerebrum into lobes began the science of localization (Casillo et al., 2020) and from there successive advances led to continuous refinement and the further breakdown of the lobes into regions. Microscopic techniques have been continuously refined, most recently through advances in light sheet imaging, two-photon imaging, polarized light microscopy, viral vectors, RNA sequencing (Zeisel et al., 2018), and many other techniques that are advancing our knowledge of the underlying neuroanatomy of the brain (Rockland and DeFelipe, 2016; Bhaduri et al., 2021; Motta et al., 2019; Gouwens et al., 2019).

Microscopic studies of cells led to the realization that the distribution of cells (type and density) varied across brain (Ecker et al., 2017). This work has focused on developing, validating and scaling emerging genomic and anatomic mapping techniques to create an inventory of neural cell types and their connections (Ecker et al., 2017). Some of the most detailed cell mapping to date has come from mouse studies where the synaptome has been cataloged and reported (Zhu et al., 2018). Work from the Allen Institute has identified the synaptic connectivity of all the inhibitory neurons in a small section of mouse tissue (Schneider-Mizell et al., 2025). Such work has provided wiring diagrams showing, for example, more than 70,000 synapses in less than a cubic mm of tissue. Whole-brain modeling of connectivity maps at the level of 100 μm voxels have been derived from such data (Knox et al., 2019). Results from the FlyWire connectome have shown a complete neuronal wiring diagram of the whole brain identifying 5 × 10^7^ chemical synapses between 139,255 neurons (Dorkenwald et al., 2024). This work highlights the complexity of the brain by showing that each individual neuron has numerous presynaptic and postsynaptic partners involving some 15.1 million connections between the 139 k neurons (Schlegel et al., 2024). A notable finding is that 93.3% of the neurons are in “strongly connected networks” (connected to more than 1 other neuron) and that many paths connect neuron pairs (Lin et al., 2024). In other words, the brain has very robust interconnectivity. These connections do not define the functional units of neural computation but instead provide a template for information processing and the development of computational models that relate complex structural pathways to function. Similar studies are underway to map the mouse brain (Ngai, 2022; Jefferies et al., 2023). While these studies are tremendously exciting and derive from elegant experimental techniques they also point to the limits of structural mapping in two senses. The first has already been mentioned - that any neuron has a pathway to any other neuron and often many paths. Secondly, such studies do not provide information on the molecular nor the functional connectome. This additional information is required to understand how neural populations work together to execute functions. Neurotransmitter signaling and a wide range of input/output variables propagating through excitatory and inhibitory pathways ultimately determine function. Cellular studies provide the organizing principles for understanding cortical wiring and the neurobiological basis for understanding function.

### The emergence of atlases: grouping cells into regions

Historically, as studies of cell types and their distributions continued, we observed the emergence of maps defined by these cell characteristics. Atlases were created based on cytoarchitecture (Amunts et al., 2020; Brodmann, 1909) with the Brodmann atlas (Brodmann, 1909) being one of the earliest. As the development of atlases has evolved, multimodal data and additional criteria have been introduced to attempt to more rigorously define cortical areas beyond simply cytoarchitecture. For example, the so-called FACT categories (Van Essen and Glasser, 2018), were introduced to incorporate *function, architecture, connectivity* and/or *topographic* organization in defining regions. *Function* in this scheme, refers to the function of a cell, not the function of the region, or how the cells fine-tuning within the region depends upon the specific inputs/output conditions. To date no consensus atlas has emerged in any species. In macaques, for example, where extensive cell and tracer studies are available, there are at least six atlases enjoying common application (Van Essen and Glasser, 2018). In further extensions, multimodal atlases have been introduced, including both animal and human atlases linked to gene expression (Yamamori and Rockland, 2006). One challenge with such neuroanatomic approaches is that the boundaries outside of primary sensory/motor areas are not clear. A bigger problem is that such regional definitions do not account for *local tuning* and how neurons *function as a group*.

The cortical layers add additional complexity in atlasing and these sometimes highlight the mismatch between different aspects of neural cells (Palomero-Gallagher and Zilles, 2019). Multimodal analyses of neural cells are now possible, and it is clear from such studies that there are cases where the borders of the cyto- and myelo- architectonic layers are compatible but the receptor density profiles (potentially more directly tied to function) rarely coincide with the histologically defined borders of layers. Even at the level of the cortical column there is a mismatch between structure and function with columns not capturing the granularity of the vertical and lateral interdigitation of the component neurons in a circuit (Douglas and Martin, 2007).

Nevertheless, interest in developing atlases continues to grow and many atlases have been introduced with the number of regions in humans ranging most commonly from 100 to 400 but sometimes as many as 5,000 (Dadi et al., 2020; Salehi et al., 2020). Early, commonly used anatomic atlases include the Anatomic atlases (AAL) (Rolls et al., 2020), freesurfer (Fischl, 2004), and others (Kaltenmark et al., 2020; Desikan et al., 2006). These early atlases are spatially coarse (similar to the Brodmann atlas) with of the order of ~100 regions or less and are being replaced by atlases in the 300–400 node range.

The decomposition of the brain into regions can be performed at any scale, from lobes, to regions, down to a single neuron. Such decompositions provide a useful basis for analysis of macroscopic activity and a framework for localizing results. However, it is important that the inherent assumptions of such decompositions are noted. While the individual neuron is the appropriate size for a fixed atlas, it is currently not possible to simultaneously study the functional activity of every neuron. Thus, atlases are used to reduce the number of dimensions by grouping many neurons into regions. Other factors to consider in the application of an atlas is the effect of brain state (Greene et al., 2023), development stage, sex, aging, and the presence or absence of neurological or neuropsychiatric disorders. See for example Eickhoff et al. (2017), for a review of these different considerations.

### Mesoscopic structural imaging—anatomic features and diffusion-based MRI

At the noninvasive *in vivo* mesoscopic scale, mapping of white matter circuits is achieved using diffusion tensor imaging (DTI) (Basser et al., 1994) or diffusion weighted imaing (DWI) methods such as HARDI (Tuch, 2004) and NODDI (Zhang et al., 2012). These MRI techniques exploit the fact that water (visible to MRI) preferentially diffuses along the axonal fiber pathways. By measuring the diffusion of water in multiple directions, the principal diffusion directions can be determined and the primary component of diffusion in the white matter is along the axonal tracks, thereby providing insight into fiber orientation (Conturo et al., 1999). Diffusion MRI can map the major white matter pathways throughout the brain with exquisite levels of detail across species (Shen et al., 2019; Feinberg et al., 2023) and has been successfully applied in post-mortem brains at very high resolution (Yendiki et al., 2022). As the MRI technology develops both anatomic and functional data are being acquired *in vivo* at higher and higher spatial resolutions (Edlow et al., 2019). Structural connections at the macroscopic scale leave open the question: “How do these circuits relate to function?.” To answer this question, measures of function are needed.

### Mesoscopic functional imaging - task based localization

Since the introduction of functional imaging via PET, NIRS and functional MRI, there has been tremendous growth in functional localization studies (Poldrack and Farah, 2015). Functional studies reveal a much more complex landscape of function than observed with cytoarchitecture. Task-based fMRI shows that the spatial extent of functional regions varies with statistical threshold, but reproducible results are clearly achievable. The size, shape and center of mass of functional regions, can be influenced by brain state (Greene et al., 2023; McKeown et al., 2025; Rugg and Thompson-Schill, 2013), and the conditions present during a task (Greene et al., 2023; Pauli et al., 2016; Assem et al., 2024; Smallwood et al., 2021). There are few studies linking task-based fMRI regions to anatomic atlases, typically because the functional imaging measures do not line up neatly with anatomic boundaries (King et al., 2019).

NeuroSynth (Yarkoni et al., 2011)-style analyses of coactivation patterns, have shown gradients of activity and provided sub-regions within structures such as the striatum (Pauli et al., 2016) and caudate. In many cases task specific brain areas have shown overlapping boundaries (Völlm et al., 2006; Hagler and Sereno, 2006) or networks (Völlm et al., 2006; Xu et al., 2013), and demonstrated a disconnect between structural and functional subdivisions (Pauli et al., 2016). High resolution studies have demonstrated organization at the level of the columns in visual cortex (Yacoub et al., 2008) and work on layer specific fMRI has been able to delineate input vs. output layers (Huber et al., 2017; Finn et al., 2019).

As noted by Simon et al. (2004), functional activations often overlap across a range of tasks potentially because multiple tasks share abstract components of cognition. To address this arguments have been made for the development of new ontologies (Varoquaux et al., 2018; Poldrack et al., 2011) suggesting that perhaps the functional labels are wrong (Eisenberg et al., 2019) and better labels would coalesce around some not-yet-defined function. These data driven ontology approaches have shown functional regions that overlap in their distributions (Yeo et al., 2016) thus returning us to subtle tuning of subsets of ensembles of neurons as an explanation for variable functional boundaries.

Distinct patterns of organization within regions have been observed brain wide. Multivariate pattern analysis (MVPA) operates at the voxel level on fMRI data (Norman et al., 2006) or sensor level in EEG/MEG data with the underlying notion that specific patterns of activity reflect specific cognitive operations. In imaging, the net result of such analyses is to demonstrate intricate functional organization at the level of voxels, revealing fine-tuning of ensembles of neurons that yields a topography specific to the stimuli (Peelen and Downing, 2023; Woo et al., 2014; Haxby et al., 2001; Haxby, 2012). It can be hypothesized that high resolution cell-based studies should also demonstrate this effect, given the small-world properties of brain organization (Bassett and Bullmore, 2006). In sum, there is clear evidence for organizational specificity within regions often labeled as a single area (Haxby et al., 2001), and fMRI studies have shown flexible boundaries dependent upon the particular task conditions.

### Meso- to macro-scopic functional connectivity—the connectome(s) and parcellation(s)

The development of brain atlases has seen renewed interest as the field of functional connectivity has expanded (Biswal et al., 1995). Network level analyses of the brain require nodes, often defined from atlases imposed upon the brain. Functional connectivity data provides a framework for developing parcellations based on the functional connections between voxels (Eickhoff et al., 2017, 2018). Parcellations to define atlases have been based on functional connectivity data alone (Craddock et al., 2012; Shen et al., 2013; Fan et al., 2016; Gordon et al., 2014; Schaefer et al., 2018), DTI data alone (Alemán-Gómez et al., 2022), or these measures combined (Fan et al., 2016; O'Muircheartaigh and Jbabdi, 2018). Other approaches include task-based fMRI, and other anatomic features such as myelination in the development of atlases in both humans (Glasser et al., 2016; Fischl and Sereno, 2018; Parisot et al., 2017) and other species (Liu et al., 2018). The multimodal parcellation approaches have assumed that joint parcellation of different types of information can yield better regional definitions (Glasser et al., 2016; Parisot et al., 2017; Pijnenburg et al., 2021) but this is not necessarily true.

Functional connectivity parcellations under different brain states has revealed flexible node reconfiguration as a function of task or brain state (Salehi et al., 2020; Greene et al., 2023). This reconfiguration is reliable enough to enable prediction of the task condition under which the parcellation data was collected. A criticism of this work has invoked a sort of Butterfly-effect argument (from chaos theory of Lorenz, 1963) wherein a small change in one region due to a task, could lead to large scale reconfiguration of the entire parcellation, suggesting reconfiguration is a parcellation algorithm problem not true functional organization. However, extensive changes in connectivity patterns have been observed within fixed atlas nodes (Ryyppo et al., 2018; Luo and Constable, 2022), and like the reconfiguration of the entire atlas, within node changes are sufficiently robust to develop predictive models that identify the conditions under which the data was collected. This is clear evidence of flexible functional organization, with function defined here as different subsets of neurons acting together under different conditions.

The focus of much connectivity research has been on how connections between nodes functionally vary with different tasks, brain traits or states (Nomi et al., 2017). Numerous studies have demonstrated flexible network (Luo et al., 2021) level organization across brain- or task- conditions using nodes defined by fixed atlases (Liao et al., 2017; Bassett and Bullmore, 2009; Chen et al., 2021). To the detriment of the field within-node changes are ignored and it has been shown that the findings reported in applications of graph theory measures across task or clinical conditions would change if within-node functional organization was considered in addition to the commonly reported between-node changes (Luo and Constable, 2022; Luo et al., 2021).

### Small world properties across scales and flexible functional considerations

The lack of consideration of functional connectivity changes within a node is inconsistent with the wide-spread acceptance of the theory that the brain exhibits small world properties (Bassett and Bullmore, 2006). This theory suggests that changes between nodes, should also be reflected at smaller scales with smaller “node” elements—say voxels (Luo and Constable, 2022) and indeed this is the case. Nevertheless, between-node changes are privileged in the literature relative to within-node changes. Imposing an atlas and measuring changes between nodes endorses the implicit assumption that all the neurons within a node have the same function—but this is not supported by the data (Luo and Constable, 2022). Avoiding the atlas problem entirely, work by Calhoun et al. (2014) has shown dynamic changes in connectivity and time-varying spatial properties of areas that couple—indicating flexible functional organization - without making *a priori* assumptions on fixed brain regions.

### Microscale dynamics and flexibility

Recent work in dynamics has shown that within a fixed synaptic architecture, dynamic network connectivity can change rapidly via the actions of neuromodulators that rapidly and flexibly alter efficacy of synaptic connections (Arnsten et al., 2012, 2024; Kringelbach et al., 2020). Understanding the microarchitecture can help to close the mechanistic gap as to how the brain executes function (Paquola et al., 2022). Flexibility manifests at different spatial scales—from individual neurons (Tao et al., 2019), to cortical ensembles (Salehi et al., 2018, 2019, 2020; Cardin, 2019), and across different time scales—from moment to moment (Rosenberg et al., 2015, 2020) changes to changes across development (Puścian et al., 2020), and learning (Vardalaki et al., 2022; Yaeger et al., 2024; Bassett et al., 2011). There is also well documented dynamic flexibility at the network level (Salehi et al., 2019; Kirst et al., 2016, 2017; Cole et al., 2013). Clearly, even at the level of a cell, fixed structure can support flexible function arising through different neural tunings, neuromodulators, and interactions within the complex neural organization of a brain.

### Mesoscale dynamics and flexibility

Much has been learned about flexibility of functional organization at the network level (Salehi et al., 2018). At the macroscopic level the brain has clear modular functional organization (Sporns and Betzel, 2016) that can be observed by integrating connectivity over time. If we look at dynamics however, we see that the modular structure is never present at any given time (Sporns et al., 2021) but dynamics provide a link between structure and function (Avena-Koenigsberger et al., 2017). Arguments have been made for more comprehensive studies of the dynome (Kopell et al., 2014)—the study of the dynamic structure–function relationships that lead to behavior. Brain modularity arises from a net integration of flexible modes over time. The macroscopic characterization of the flexible representation of connections between nodes is relatively easy to measure, and hence the literature is full of such studies. Yet there is no reason to believe these organizational principles do not extend to the meso-(Zeng, 2018; Li et al., 2019) and micro-scopic scales, where connectomics and networks are still found (Bassett and Bullmore, 2006).

### Microscopic to macroscopic functional flexibility

There is clearly a fixed anatomic infrastructure which is sparse relative to the much more complex functional architecture (Bullmore and Sporns, 2009; Sporns, 2013). On top of this fixed neuroanatomic basis sits a complex functional organization that both cell-based studies, and neuroimaging studies demonstrate is quite flexible. This flexibility occurs as different input/output brain-state conditions lead to different subsets of neurons to fire together to execute a task (Reich et al., 2001; Wu et al., 2021). In this sense there is no single atlas that defines a functional region (Salehi et al., 2020). There is no functional equivalent to the Brodmann atlas based on cytoarchitecture. Instead, there are infinite combinations of neurons that may work together under different conditions. This flexibility should not be overlooked by a singular focus on anatomic atlas definitions.

### Caution in how brain atlases are applied and other considerations

The human brain contains 80–100 billion neurons (Matsumoto et al., 2024; Herculano-Houzel, 2009; Williams and Herrup, 1988) and dividing these into 100 or even 1,000 areal regions places millions of neurons in any given region. Philosophically, no one would argue that the level of redundancy in the brain is such that the millions of neurons in a region all do the same thing. However, in the application of atlases, this is exactly the assumption made. If we are to make advances in understanding brain function, we need to consider the underlying complexity and find ways to understand the different levels of organization these millions of neurons support.

Functional definitions based on cytoarchitecture are not the same as functional regions defined as ensembles of neurons. This is where there is currently a disconnect in the field. Consider the visual cortex. V1 is a clearly defined both via cytoarchitecture and through functional stimulation, but it also clearly has many levels of functional organization (Kumar et al., 2021; Stringer et al., 2021). Similar levels of organization are found in MT (Van Essen and Glasser, 2018), and all other areas of the brain ranging from motor (Gordon et al., 2023) to frontal cortex (Gilbert et al., 2010). The organizing principles become much more difficult as we move to higher cortical regions, and this mismatch between cellular level function and ensemble activity increases.

Simply working at the voxel level does not avoid the atlas problem—it just changes the scale. A voxel is still a parcellation, in this case defined by the acquisition scheme, and a single fMRI imaging voxel can contain 100’s of 1,000 of neurons—not all of which may have precisely the same tuning. Data from the Allen Institute has demonstrated that even in one cubic millimeter of tissue there is complex organization (Schneider-Mizell et al., 2025). The ultimate resolution in which ensemble activity appears to be fixed and structure and function match more closely is the level of the neuron. Any atlas above this level is open to selective activation of subsets of neurons according to the specifics of the task, the cell tuning, and the molecular environment.

Methods such as independent component analyses (ICA)(Calhoun et al., 2001) avoid imposing an atlas on the data but still end up partitioning the data into (sometimes non-spatially-) contiguous components for analyses. These methods yield far fewer components that is typically encountered with parcellation analyses; often with less than 100 components considered. Nevertheless, this atlas-free approach has shown flexible temporal/spatial organization of these components in the brain (Xu et al., 2013; Allen et al., 2012) supporting the notion of flexible functional organization on top of a fixed underlying structural organization.

Another data driven approach to understanding brain organization has come from the analysis of gradients of connectivity and function across the cortex. Strong evidence of hierarchical organization in terms of gradients of function (Posani et al., 2025; Margulies et al., 2016; Leech et al., 2023) have been shown, and these gradients place the default-mode network along principal axes of organization. Work examining gradients of structure–function coupling across the cortex has shown close links in unimodal, primary sensory and motor areas but much more divergence in transmodal cortex (Vázquez-Rodríguez et al., 2019). This approach links the topological organization of the brain to the functional organization without the need for defining a fixed atlas.

### Moving forward/how and when to accommodate flexible functional organization

At a minimum, atlases represent a useful data reduction strategy necessary for many analyses. Analytic approaches such as brain-behavior modeling are generally not sensitive to the choice of atlas in the range of ~200–400 nodes. This is because in building the models there is a data reduction step when all the edges above some feature selection threshold are summed to yield a network score (Shen et al., 2017). This reduces the data from often over 1,000 edges, to a single network score (usually the sum of z-score edge values). Such summary statistics are mostly insensitive to the choice of atlas. If nonlinear methods are used such as Kernel Ridge Regression, feature selection is not applied and all the 35,000–70,000 edges in the connectivity matrix (dependent upon the atlas imposed) are included in the model. With this scale of analysis (200+ node range), the results do not appear to be sensitive to the specific atlas used (Messé, 2020).

For most analyses it appears that a balance between node homogeneity and intersubject anatomic/organizational variance lies in the 200–400 node range for many applications in fMRI. Larger atlases have been developed and it has been shown that node reconfiguration is still sufficient to predict brain-state in atlases with up to 5,000 nodes (Salehi et al., 2020). This suggests that simply moving to a much larger atlas does not solve the node (re)organization problem and this undoubtably holds until one is at the level of the single neuron. There is still a need for a common space (Devlin and Poldrack, 2007) for reporting results but rather than atlas based reports, the continued use of Talairach and Szikla (1979) and Lacadie et al. (2008) for center-of-mass of functional regions should be used.

## Summary

Throughout the history of neuroscience there have been evolving concepts of functional localization (McCaffrey, 2023). The gap between single neurons and ensembles of neurons remains to be filled but embracing concepts of sub-specialization (or tuning) of neural cells in any region, combined with modeling concepts that rely on subsets of neurons (Papadimitriou et al., 2020) should lead to further progress in understanding brain function. While it is essential to map the underlying infrastructure of the brain, it is also essential to recognize that this architecture supports flexible functional organization. Constraining flexible functional organization by imposing fixed atlases should be avoided if possible and at a minimum the assumptions of fixed atlases in the presence of flexible organization should be acknowledged as the field moves forward (Gamlin et al., 2025).

## Data Availability

The original contributions presented in the study are included in the article/supplementary material, further inquiries can be directed to the corresponding author/s.
